# The roles of men and women in maternal and child nutrition in urban South Africa: A qualitative secondary analysis

**DOI:** 10.1111/mcn.13161

**Published:** 2021-03-10

**Authors:** Agnes Erzse, Susan Goldstein, Aviva Tugendhaft, Shane A. Norris, Mary Barker, Karen J. Hofman, for the INPreP group

**Affiliations:** ^1^ SAMRC/Centre for Health Economics and Decision Science, PRICELESS, School of Public Health, Faculty of Health Sciences University of Witwatersrand Johannesburg South Africa; ^2^ SAMRC Developmental Pathways for Health Research Unit University of the Witwatersrand Johannesburg South Africa; ^3^ School of Human Development and Health, Faculty of Medicine University of Southampton Southampton UK; ^4^ MRC Lifecourse Epidemiology Unit, University of Southampton and NIHR Southampton Biomedical Research Centre University Hospitals Southampton NHS Foundation Trust Southampton UK

**Keywords:** gender, maternal and child health, nutrition, qualitative secondary analysis, South Africa

## Abstract

Industrialization and urbanization processes have challenged deeply held traditional gender norms and facilitated the emergence of modern ideologies in South Africa. This paper seeks to explore the gendered roles of family members on maternal and child nutrition and investigate indications of perceived change in related practices. A qualitative secondary analysis was conducted of data from nine focus group discussions (FGDs) held with men (*n* = 3) and women (*n* = 6) aged ≥18. Data from the FGD were coded and thematic analysis conducted. We found that elderly women seem to have a central advisory role with respect to maternal and child nutrition and that men and elderly women upheld patriarchal gender divisions of labour, which entrust mothers with the primarily responsibility for young children's nutrition. Young mothers relied on elderly women for provision of childcare and nutritious foods for children; however, they demonstrated some resistance to traditionally feminized forms of food preparation. We found that men's involvement in children's nutrition was limited, though they expressed a preference to be more involved in maternal and child nutrition and care practices. A gender transformative approach to policy development, which includes elderly women and men, has the potential to promote more gender‐equitable nutrition practices, increase young women's self‐efficacy and support them to overcome barriers that could be limiting their decision making power in achieving optimal nutrition for themselves and their children.

**Key messages**

- In Soweto, a rapidly urbanizing area, young women's and men's attitudes appear to be changing towards maternal and child nutrition.
- Policies to improve maternal and child nutrition should attempt to challenge and transform existing power relations and become more gender equitable.
- Strengthening young mothers' agency in nutrition decision making and engaging men in becoming role models for healthy eating from early ages should be a priority.
- To ensure gender transformative approaches in early life nutrition, we should consider men's and elderly women's role in reinforcing or changing present gender inequities in nutrition‐related practices.

## 1 INTRODUCTION

South Africa's post‐apartheid democratic reform agenda is one of the most progressive in the world. The achievement of equality is a foundational value of the Constitution of 1996, granting the right to equality, dignity, culture and language (‘Constitution of the Republic of South Africa (Act No. 108 of 1996)’, 1996). South Africa has reached gender parity with respect to school enrolment with increases the numbers of women in ministerial positions (48%) and one of the highest proportions (46%) of female members of parliament in the world (World Economic Forum, 2019). Findings from the South African Social Attitude Survey (SASAS 2012) also paint the picture of an increasing degree of feminization of the labour market and shifting of conventional gender models (Human Sciences Research Council, 2014). The majority of respondents (88%) agreed that both men and women should contribute to the household income. However, this positive attitude towards women entering the work force has not necessarily led to the social empowerment of women or greater equity in the household with housework and childcare; they continue to be influenced by conventional patriarchal gender models. Prevailing attitudes include the idea that women should be responsible for shouldering the burden of grocery shopping, preparing meals and caring for children. One in two men and women believed that during the first few years of a child's life, the most critical time period for children's short‐ and long‐term health, growth and development, the mother should stay at home and the father should work full‐time (Human Sciences Research Council, 2014).

Progressive gender equality laws in South Africa introduced since 1994 also parallel processes of industrialization and urbanization. These transitions challenge core values around gender norms within the households; rapidly emerging modern ideologies clash with persisting traditional cultural values (Cohen et al., 2019). On the one hand, women are perceived to comply with a patriarchal attitude to household tasks, while an emerging sociocultural drive to achieve individual emancipation is increasingly recognized among new generations of women (Cohen et al., 2019).

Modernization has introduced changes to the food system including the nutrition transition to ultraprocessed foods across the globe and South Africa is no exception (Nnyepi et al., 2015). It has turned urban areas into dynamic frontiers where traditional and modern nutrition practices overlap and intermesh densely (Kroll, 2016). On the one hand, a modernist ideology of individualism, with symbols of economic advancement is marketed and internalized with respect to fast foods, sugar‐sweetened beverages, ultraprocessed snacks and sweets (Kroll, 2016). On the other hand, commodification of foods, in other words treating food as a mere commodity, obscures non‐economic values and dimensions of nutrition such as food as a symbol of culture or human rights (Vivero‐Pol, 2017). At time when food is created within a contemporary commodity culture, there is a reason to believe that deeply held values and notions of identity linked to food are altered (Lewis, 2016). It is, however, unknown if and how gender roles have changed in the context of this nutrition transition.

This study aimed to investigate the gender dynamics and roles of family members with respect to maternal and child nutrition (MCN) in an urban setting in South Africa. The objectives were (1) to identify the roles of and influences on men and women in delivering MCN and (2) to identify perceived changes to practices linked to MCN.

This research has implications for designing policies and programmes targeted MCN in transitioning populations.

## 2 METHODS

### 2.1 Qualitative secondary analysis

The study is a qualitative secondary analysis (QSA) (Heaton, 2008) of data from focus group discussions (FGDs) carried out as part of a study involving urban communities in Soweto, South Africa. QSA uses pre‐existing data derived from previous studies to investigate new research question without further burdening respondents (Heaton, 2008). We approached QSA as investigators who were interested in a concept that emerged but not fully explored in pursuing the objectives of the primary study (Erzse et al., 2020). The primary study suggested that MCN practices and perceptions of participants followed certain socially entrenched rules of behaviours; this generated new questions about gendered aspects of MCN. We are exploring two new research objectives in this QSA:

1. to investigate the roles of men and women and their influences on MCN within the household and
2. to identify indications of perceived change to practices linked to MCN.

All authors except one were involved in the primary study. We have invited one additional author with extensive qualitative research experience but who was not involved in the primary study. This was done to obtain inputs that are not influenced by involvement in the primary study and to strengthen the scientific rigour of the QSA.

### 2.2 The primary study

The primary study was a piece of qualitative research using FGDs and which was designed to explore community perceptions of MCN issues during the first 1,000 days of life and their recommendations for solutions in Soweto, a large urban area in southwest of Johannesburg. Soweto was selected as the study site to harmonize with other work relating to nutrition and the first 1,000 days carried out locally. In Soweto, the nutritional status of children and women is poor with high rates of stunting (34%) among children under 2 (Nyati et al., 2019), and overweight and obesity (66%) and anaemia (31%) among women (Prioreschi et al., 2017). The data are ideal for the QSA presented in this paper because as in other urbanizing communities, the area is undergoing significant socio‐economic change (City of Johannesburg, 2018). This includes the growing prevalence of a more Westernized diet that is higher in energy, salt, sugar and saturated fat. Soweto's food environment is characterized by numerous commercial and informal food outlets (i.e., tuck shops and street vendors) selling energy‐dense, fast‐food products.

### 2.3 Primary study procedures

Primary data were collected through nine FGDs, three with men aged 18 and above and six with women, two with women aged 18–25, two with older women aged 26–40, and two with women above the age of 40. Participants were purposefully recruited from those waiting at the main transportation hub in the study area between February and March 2019; they were not known to each other. FGDs were moderated by two fieldworkers with extensive qualitative research experience. Prior to data collection, the fieldworkers received training in using the study protocol, including the semi‐structured interview schedule that guided the FGDs. The FGDs were run until thematic saturation was achieved in meeting the objective of the primary study (Fusch & Ness, 2015). Thematic saturation in relation to the focus of the QSA was not a realistic aim because the data were not collected for this purpose. Discussions were conducted in a mixture of participants' home languages and English, and they were digitally recorded. An independent transcriber transcribed and translated each discussion verbatim, and transcripts were checked against the recordings before data analysis. All participants provided written consent prior to the FGDs. The consent forms also secured consent for future use of data for research purposes, including the QSA.

### 2.4 Primary study participant characteristics

A total of 45 women and 21 men, age 18–67 (mean 34) participated in the primary study. We present their sociodemographic characteristics in Table 1. Of the respondents, 47% attended high school, and 21% had obtained a high school diploma (matric). The majority of men and women were single or never married, results that align with those in the 2016 South Africa Demographic Health Survey where 59% of women and 65% of men have never been married or lived with a partner (National Department of Health (NDoH) et al., 2019). Most participants (91%) cared for one or more children. Of the men, three indicate that they had no childcare responsibilities. Fifteen percent of all respondents were in formal employment and 79% lived in low‐income households with less than ZAR 5000 a month. In 2019, the national upper bound poverty line was ZAR 1227 per person per month (Statistics South Africa, 2019b). Forty percent of women relied on government grants for their main source of income, and 67% of men reported employment in the informal sector, meaning that they sold goods and provided services to make just enough money to meet their family's daily needs.

**TABLE 1:** Sociodemographic characteristics of primary study participants

Characteristics	Men (*n* = 21)	Women (*n* = 45)	Total (*n* = 66)
Age (years), mean (SD)	32 (9)	35 (13)	34 (12)
Marital status		*N (%)* ^*^	
Single	14 (67)	24 (53)	38 (58)
Married	2 (10)	8 (18)	10 (15)
Living together	1 (5)	8 (18)	9 (14)
Divorced/widowed	3 (14)	4 (9)	7 (11)
Unknown	2 (10)	0	2 (3)
Caring for at least one child	18 (86)	42 (93)	60 (91)
Education level			
No formal education	0	1(2)	1 (2)
Complete primary	15 (71)	20 (44)	35 (53)
Complete secondary	4 (19)	10 (22)	14 (21)
Higher than secondary	2 (10)	13 (28)	15 (23)
Missing data	1 (5)	0	1 (2)
Monthly household income			
Less than ZAR 3,000	14 (67)	29 (64)	43 (65)
ZAR 3,001–5,000	3 (14)	6 (13)	9 (14)
More than ZAR 5,000	3 (14)	6 (13)	9 (14)
Unknown	1 (5)	4 (9)	5 (8)
Source of income			
Formal employment	4 (19)	6 (13)	10 (15)
Self‐employed	14 (67)	6 (13)	20 (30)
Others^**^	3 (14)	27 (60)	30 (45)
Unknown	0	6 (13)	6 (9)

^*^ Percentages do not add up to 100 due to rounding off.

^**^ E.g., Government grants.

Source: (Erzse et al., 2020).

### 2.5 Data analysis

QSA was completed after findings from primary study were published. Existing transcripts (*n* = 9) from the primary study data set were uploaded into Nvivo version 12 (NVivo qualitative data analysis software, 2020). Clean transcripts were independently coded by two researchers (AE and SG). For the primary study, coding guides were developed in alignment with the interview guides. For the QSA, researchers categorized the transcripts by gender and age and developed deductive themes against which to code the data. These included the roles and influence of *women*, *elderly women*, *men* in nutrition, and *indications of perceived changes to norms*. To capture change, we searched for references in the transcripts to intergenerational difference between the groups of women of different ages. Because the sample did not allow the same approach to be taken to with the groups comprised of men, indications of change were identified through a comparison of men's aspirations with their current practices. Our aim was to discuss the diversity of experience and opinion among the groups of men and women we spoke to rather than to report on the generalizability of our findings.

Reporting of the findings adheres to COREQ guidelines (Tong et al., 2007).

### 2.6 Ethical consideration

The primary study was conducted according to the guidelines laid down in the Declaration of Helsinki and all procedures involving research study participants were approved by the University of the Witwatersrand Human Research Ethics Committee (Medical) (Clearance certificate number M181056) and the University of Southampton (47290). Ethical approval was not required for the QSA of the data in the present paper.

## 3 RESULTS

Thematic analysis of the nine FGD transcripts identified critical themes in participants' discussions of the roles and influence of men and women in MCN and in indications of perceived change to practices. Data as they apply to these themes and meeting the objectives of the study are presented below.

### 3.1 Objective 1: The roles of men and women and their influence on MCN

Emerging themes included mothers as implementers of daily domestic tasks, preparing food, caring for children and carrying out income‐generating activities; elderly women as supervisors and advisors of women on nutrition, supporting women in raising children, preparing meals, feeding children, and promoting vegetables within the households; men as head of households and as providers, promoting healthfulness in and around the households, their patriarchal attempts to monitor women, and a contrasting of men who participate in childcare and ‘irresponsible fathers’ who do not. The roles played by various actors in relation to MCN are detailed in Table 2 with illustrative quotes.

**TABLE 2:** Roles of mothers, elderly women and men in maternal and child nutrition

Population group	Roles related to maternal and child nutrition	Illustrative quotes from FGDs
Mothers	Implementing daily domestic tasks to support family life	‘Women are the ones that are keeping their households together, so when you wake up in the morning and the child wants food, they want it from you. The father is there ‐ maybe he's not working but the child won't ask the father for food, they'll ask you’. (Women, 26–40, FGD 1) ‘You're failing as a mother, […] you're expected to be everything, you must be strong, you must know everything, and they forget that you are a first time mom’ (Women, 26–40, FGD 1)
	Preparing food for the family	
	Caring for children, e.g., feeding children, taking sick children to clinics	
	Carrying out income‐generating activities	
Elderly women	Supervising and advising women before, during and after pregnancy	‘I used to drink Fanta wherever I was and my grandmother was like “Hey stop drinking this thing. Your child will have jaundice”’. (Women, 40+, FGD 8) ‘With my daughter, I even had to go with her, when she saw her belly, she was approaching 4 months I said we're going, and when she tried to protest I was like no, we're catching a taxi and going, tomorrow morning we're going to the clinic’ (Women, 40+, FGD 6)
	Caring for new‐borns and young children	‘They grow up properly if there is an adult like, if you have your mother and your grandmother. Your grandmother is able to tell you what a baby needs, if you’'re alone with no adult, it's difficult because a man will not help you with anything’. (Women, 26–40, FGD 2) ‘I used to work but I don't work anymore, I live with my kids, four kids; so I have to hustle, like sometimes I'll go to my granny to help me so that my children can eat. My partner doesn't work’ (Men, FGD 5)
	Advising teenage girls on nutrition	‘When you start being a teenager, especially when you're a girl, that's when they *[elderly]* start restricting you. I used to eat boiled eggs and then my mother said I should stop’. (Women, 18–25, FGD 4)
	Advising on breastfeeding and complementary feeding	‘That time my child is healthy and has the right weight, but they *[elderly women]* said milk is not enough, because they used to feed me when I was 3 months so my child also needs to be fed … and it's very difficult to remain still as a parent and say no I want to do this for my child, so it was the main problem I had, and I eventually I think I gave in when he was 5 months, they started feeding him porridge’. (Women, 26–40, FGD 1) ‘Immediately when they turn 3 days … grannies will tell you about porridge … so that the baby can sleep’. (Women, 26–40, FGD 2) ‘When she was crying incessantly, my mother, my grandmother gave her 2 spoons of porridge. That's when I realised that it's hunger’. (Women, 26–40, FGD 2)
	Preparing meals for and feeding of young children	‘My mom also used to buy veggies like squash, butternut and make it for her *[my child]*, potatoes. She would say it's for my son Nkosana, not for our consumption for supper meals’. (Women, 18–25, FGD 3)
	Promoting and providing vegetables in the household	
Fathers	Providing resources for family functioning and well‐being	‘You come to the table, you come with bread, what I mean by bread is the income you've earned, then you'll be able to provide the basic needs that the family will want, basic food stuffs you know, which is healthy’. (Men, FGD 7)
	Caring for mothers and children	‘Fathers need to start looking after their kids, without the father contributing money the child cannot get that type of food he or she needs to eat’. (Men, FGD 9) ‘Buy food that's good for someone that's pregnant, and the environment where they live needs to be 100% *[healthy]*; that is your responsibility as a man’. (Men, FGD 5)
	Monitoring women	‘I'd make sure that the child's procedure for eating, she *[mother]* follows it regularly to the point when they are meant to be fed’. (Men, FGD 9) ‘We need to see how they *[mothers]* cook at home, because you can't be putting too much oil whereas the oil itself is not healthy oil’. (Men, FGD 9) ‘It's also our job just to remind them, you see, keep a watchful eye, because we also tend to ignore pregnant women and assume that she already knows what to do. So when you are able to as the father, you need to remind her if she's taken her medication, or this and that; then it will help’. (Men, FGD 9) ‘She can eat the whole day but as long as they eat the right food not junk food, and they must also drink water’. (Men, FGD 5) ‘The mother needs to eat veggies so that the child can grow big, you understand, and that very child needs to eat veggies when they're older’. (Men, FGD 5)
	Promote healthfulness in and around the household	‘That child needs to grow up knowing that *[…]* every night their plate must have a veggie. You as the man, the father need to install that system’. (Men, FGD 9) ‘In my neighbourhood we make sure that we keep them clean. We also place boards that say “no dumping”; they're still going to dump but we make it a point’. (Men, FGD 9)
	‘Irresponsible fathers’	‘It's difficult because a man will not help you with anything, he'll leave you with your child, and you'll crack your head trying to make it work’. (Women, 26–40, FGD 2) ‘Starving your child the whole day and when you get home and maybe you've left the child with their father; and then when you get home the child is not playing, they're just malaise basically, so … when you get there and ask did the child eat [...] and he's like no, she didn't say she's hungry’. (Women, 18–25, FGD 4) ‘Provide at home first and then you can leave some money behind for yourself to go enjoy with your friends and buy that alcohol, but you find that guys spend all that money recklessly and then end up going to loan sharks, they don't even have money to go to work’. (Men, FGD 9)

Abbreviation: FGD, focus group discussion.

#### 3.1.1 Roles of mothers

In general, mothers were seen as responsible for optimal nutrition. They experienced a number of challenges in acquiring enough nutritious food for their children and themselves, however. Women reported financial hardship, job insecurity, difficulty coordinating school and work leaving little time to care and cook for the children. The compounding tasks left women, particularly first‐time mothers, feeling stressed and unable to manage. A general sense of hardship characterized women's discussions, with some of the most frequently used words being: help, difficult, granny or grandmother.

#### 3.1.2 Roles of elderly women

Findings suggested that grandmothers' and other elderly women's support in childcare was highly valued across all groups. Elderly women were seen as experienced advisors and supervisors of women on MCN. Younger participants perceived elderly women's advice beneficial on some occasions and restrictive in other instances. Participants explained that grandmothers played a pivotal role in providing and promoting vegetables in the household, with a priority focus on young children. Mothers were grateful that grandmothers encouraged locally available vegetables, often grown by themselves, and helped prepare meals for children.

When asked about exclusive breastfeeding (EBF), almost all women described the influence of elderly women to be disruptive to EBF practices. Participants commented frequently that elderly women in their communities believed that a crying baby was hungry and that breast milk is inadequate to assuage that hunger. Mothers were therefore told that they should feed the baby solid food.

Mothers felt conflicted and pressured to take advice from elderly women despite understanding that this may not be the healthiest option for their infants. This advice included introducing porridge in the bottle and milky tea as early as 2 months of age; this differs from recommendations for EBF of 6 months. Mothers usually followed the advice of elderly women, however, given their authoritative supervisory and supporting role.

#### 3.1.3 Roles of men

There was a difference between the men's and women's discourse. Whereas women talked about their lived experiences, men described aspiring to role as provider and controller of the household. Even where they were not currently in a position to do this, men described their wish to provide food for the family and ensure their well‐being, whilst at the same time assigning domestic tasks such as caring for children to women.

We found no indication in the data that men routinely fed children. Nevertheless, they expressed a sense of control over how women should manage their own and their children's nutrition. Men expected mothers to pursue a nutritious diet for the benefit of the child's health and set good examples for their children through healthy dietary practices. Although the data did not suggest that men consider themselves as role models, they exhibited a strong sense of responsibility for promoting health both within and outside of the household. This included emphasizing the need for habitual consumption of fruits and vegetables for children and close management and monitoring of mothers' food preparation practices in the home to ensure healthfulness. Sanitation and hygiene were a priority topic across the groups composed of men. The importance of maintaining the cleanliness of the household's living environment was a dominant topic for discussion in these groups.

These were clear contradictions between men's aspirations and the accounts they gave of their lived experiences. Both men and women described men and their actual practices as negligent. Men desired to be responsible fathers, yet in practice, they admitted to reneging on their childcare and nutrition duties. Men also shared their common experiences of other men's alcohol and tobacco consumption around children and unhygienic practices on the streets.

A summary of findings on the roles of men and women and their influence on MCN within the household is given in Box 1.

Box 1 **Summary of key findings of the roles of men and women and their influence on maternal and child nutrition (MCN)**

1. Young mothers are not independent decision makers regarding early life nutrition and their practices are greatly influenced by the advice of older women.
2. Elderly women serve as advisors, supervisors and caregivers in relation to the nutrition of women and children before, during and after pregnancy.
3. Men see themselves as the head of the households but are not directly involved in maternal and child nutrition.

### 3.2 Objective 2: Indications of perceived changes to practices linked to MCN

Discussions with study participants, all from urban communities, indicated that there were several evolving ideas and new perspectives around food and nutrition practices. Participants spontaneously referred to ‘traditional’ and ‘modern’ when discussing nutrition practices and perceptions in the context of socio‐economic transition. What they meant by modern and traditional they did not define precisely but was clearly commonly understood by them. Box 2 summarizes these evolving ideas and practices, all unsurprisingly attributed to young women and men. There were no indications that elderly women had changed their nutrition‐related practices.

Box 2 **Summary of perceived changes to practices linked to maternal and child nutrition (MCN)**

**Table:** 

Younger women: • Turn to nontraditional sources of information • Spend less time preparing food • Show less appreciation of home cooked food • Eat outside the home	Men: • Show concern about the type and quality of food consumed by mothers and children • Show concern about the healthfulness of living environment • Wish to play a bigger role in mothers' well‐being

Young women acknowledged elderly women's advice on nutrition; however, they explained that they also turn to other sources for information including the internet, the media and literature. They used terms such as ‘my generation’ (vs. older generations), ‘new’ and ‘nowadays’ suggesting a contrast with previous times.

I think within my generation, I'm someone who likes reading and researching a lot so I can try something new, and not to say that I'm not listening to my granny … But let me try this instead, I'd be more open, I don't know others, maybe they'll think granny knows better. (Women, 26–40, FGD 2)

Facilitator: You are pregnant, you have no help, you have nowhere to go for more information … where do you get more information?

Participants: Nowadays you Google. On these very phones. (Women, 26–40, FGD 2)

We found perceptions that a number of aspects of eating, including preparation, spatial aspect, social aspect and appreciation, were in the process of changing. Resistance from younger women to spending time preparing food and making home‐cooked meals was widely discussed. Elderly participants explained that younger women rely on take‐away foods for both them and their child.

They [young women] don't want to learn [to cook]. And this TV is also ruining them, “we are tired of cooking, today it's pizza". (Women, 40+, FGD 8)

You cook in the house and they won't touch it, they'll open the pot and see the spinach and tell you they're not having it. They just want to eat their fast food; they don't want healthy food in the house. (Women, 40+, FGD 8)

This was reinforced by younger women.

The thing is, when you start leaving home, you start living the junk lifestyle. (Women, 18–25, FGD 4)

When the kids get back from school and you don't feel like cooking you give them money to go buy kota [unhealthy South African street food]. (Women, 26–40, FGD 2)

Women explained that fast food is convenient at times when they have no time for cooking. Furthermore, it was felt to be cheap and tasty food that replaced tastes and memories of their childhood that they often associated with poverty.

You're still worried that people will think you're poor at home, to the point where you plant veggies, it's so bad that you need to plant food, you understand. (Women, 18–25, FGD 3)

But I have a problem, maybe because I grew up eating these things; I now find it hard to eat pap and cabbage. It's hard? It's hard because I grew up eating these things … I ate it a lot when I was young …. (Women, 26–40, FGD 2)

Data did not allow the same comparison to be made between younger and older men. Though acknowledged that they may not always have been responsible parents, men described wanting to play a bigger role in the nutrition and well‐being of mothers and their children.

When my partner came to deliver our baby, they close us outside and yet me as the father I want to come inside and see that indeed these women do suffer. I sometimes wished I could go inside the labour room and be with my partner as she's about to give birth, but I was not there. (Men, FGD 9)

Men expressed their openness to new knowledge that they might not have been exposed to before as part of their social roles.

Sometimes your partner sends you to the shops to buy all these things, milk, pads, and all that but you're dealing with your shame. There comes a time when we need to know feminine things you know, then we can be better father. (Men, FGD 9)

I would prefer to make changes with the women you know. I sometimes watch programs from overseas. You find that a woman is pregnant and she goes with her man to these classes. That's where you learn how to breathe in and breathe out and things like that …. (Men, FGD 9)

## 4 DISCUSSION

Through discussions with nine groups of men and women from Soweto, South Africa, we explored perceptions of their roles in and influence on MCN and identified perceived changes to long‐established nutrition‐related practices that were accompanying economic transition.

Gendered aspect of nutrition received little attention in previous research in South Africa. Studies on household food insecurity and agriculture (Reddy & Moletsane, 2009; Ruiters & Wildschutt, 2010), and social and cultural determinants of nutrition (Chakona & Shackleton, 2019; Madhavan & Townsend, 2007) have briefly reflected on the binary division of gender roles. Furthermore, there are few data collected from men in previous nutrition‐specific studies (Phillips et al., 2016). To our knowledge, this is the first study in South Africa that primarily focused on the gender roles in MCN from the perspective of both men and women and that challenged certain long‐established norms.

We found that elderly women still hold a central advisory position for families on issues to do with mother and child nutrition from birth, in the early years, through adolescence, and during pregnancy. Our study observed elderly women's involvement to be simultaneously beneficial and potentially harmful to the nutrition of mothers and children and their perceptions of nutrition as an exclusively women's role, to reinforce long‐held gender imbalances. Young mothers often rely on elderly women for provision of childcare and nutritious foods for children in South Africa (Horwood et al., 2019). However, our data suggest that this dependence can coincide with feelings of disempowerment and lack of agency. Young mothers felt pressured to obey the advice of elderly women on breastfeeding and complementary feeding and adhere to this advice despite knowing that it might not be optimal. These findings are consistent with those of Chakona (Chakona, 2020), and Mamabolo et al. (Mamabolo et al., 2004) in rural South Africa, where elderly women advised younger mothers to give infants water and other fluids such as herbal mixtures early in life. Our findings also showed that younger women perceived an intergenerational difference between elderly women and ‘in our generation’ who reported a tendency to move beyond older women's advice towards modern and independent information sources, including the internet.

We also found evidence of eagerness of elderly women to talk about preparing food at home. It can be assumed that traditionally feminized task of food preparation was central to elderly women's identity. This could not be said of younger mothers. Elderly women prioritized home‐cooked food, whereas younger women preferred ready‐to‐eat modern food. In general, participants discussed the concept of modern versus traditional as a multidimensional phenomenon and the co‐occurrence of multiple facets, resonating with findings in the broader literature (Sproesser et al., 2019). For example, food could be prepared in a grandmother's way (traditional), but it could be made using ingredients that are industrially ultraprocessed (modern).

The generational division in preparing home‐cooked meals versus buying modern food had also been reported in rural SA, where grandmothers perceived young mothers ‘resistant’ to prepare traditional foods (Chakona, 2020).

In an auto‐ethnography on the human experiences that food work can generate (Lewis, 2016), it is argued that women tend to acquire agency through creating relational bonds, by cooking and feeding their families, generating a sense of security, well‐being and contentment (Lewis, 2016). In our context, Lewis's observation could hold true for elderly women, less so for younger women. Our data did not suggest that younger women would acquire social recognition and feelings of empowerment through traditionally entrenched gender roles around feeding others. Although in our study both elderly women and men sustained the perceptions of nutrition as traditionally feminized roles, this did not seem to be the case among younger generation of women.

Our study found that the concept of men as the ‘household head’ remained central to men's identity. In reality, however, many South African children grow up in households with absent fathers. In 2019, 29.8% of households were female‐headed (Statistics South Africa, 2019a) and most children lived only with their mothers (43.1%), whereas a much smaller percentage (3,3%) of children lived only with their fathers.

South African studies have, however, revealed an increasing degree of interest of men in childcare since the beginning of the 21st century (Van den Berg & Makusha, 2018). Nevertheless, when thinking about nutrition of mothers and children, our findings suggest little direct involvement of men consistent with previous studies. The literature on fathers' involvement in MCN has described paternal roles as being limited to economic providers for the household (Horwood et al., 2019; Madhavan & Townsend, 2007).

However, we uncovered additional important paternal roles that men aspired to, such as monitoring the quality of the food consumed by mothers and children, limiting unhealthy foods including those high in sugar, fat and oil. Father also described wanting to better support their partner's well‐being and mental health during pregnancy.

### 4.1 Implications for public policy

This study highlights some salient aspects of women's and men's roles that appear to challenge long‐established gender stereotypes around MCN in an urban community in Soweto. Although findings cannot be generalized for the overall population, learning from the perceptions of urban community members is valuable source of information for countries undergoing similar rapid urbanization. The identified aspirations towards social changes and more equitable gender roles have also significant policy implications. SA's nutrition policies, such as the Roadmap for Nutrition (National Department of Health, 2013), are gender sensitive but are silent on the use of gender transformative approaches in nutrition. Gender transformative policies and programmes foster critical examination of gender norms and dynamics and create or strengthen systems for gender equity (Muralidharan et al., 2015). Current strategies in South Africa do not challenge nutrition‐related gender‐inequitable norms and practices. Inadequate inclusion of men in the nutrition discussions and activities (i.e., breastfeeding) could enforce the notion of child feeding and nutrition as ‘women's business’, thereby perpetuating gender inequality.

One way of improving MCN in the home may be to strengthen young mothers' agency in nutrition decision making and engage men in becoming role models of healthy eating from early ages. A systematic review of public health nutrition interventions in low‐ and middle ‐income coutnries showed that gender transformative programmes are effective in enhancing gender‐equitable attitudes, contribute to women's self‐efficacy and increase the inclusion of both partners in decision making (Muralidharan et al., 2015). Evidence suggests that couple‐focused behaviour change interventions may be more effective than those ascribed to individuals and can result in sustained lifestyle changes with positive implications for diet (Arden‐Close & McGrath, 2017). Transferability of such evidence should be interpreted with caution, given the low percentages of parents living together (33.8%) in South Africa (Statistics South Africa, 2019a). Local evidence generation on the effectiveness of couple‐focused interventions will be imperative. To ensure gender transformative approaches in early life nutrition, we cannot ignore grandmothers' role in reinforcing or changing present gender inequities in nutrition. Interventions may find success through engaging grandmothers in nutrition education that could help challenge traditional gender norms and change incorrect perceptions of nutrition behaviours. There is evidence from Senegal that when grandmothers are actively involved in public health programmes, practices related to maternal diet and infant and child feeding can substantially be improved (Aubel et al., 2004). A number of methodological underpinnings led to the success of the nutrition education intervention in Senegal, including a transformative learning method that engaged grandmothers to actively and critically analyse their own experiences and solutions for their problems and an education approach that empowered grandmothers to collectively solve problems. The intervention also acknowledged participants' nutrition‐related values, practices and their roles in the communities, facilitating the acceptance and integration of ‘modern’ ideas into grandmothers' advice around MCN. There is a reason to believe that policies to improve MCN would benefit from integrating elderly women in interventions in South Africa. In the context of high prevalence of single‐parent households and unemployment rates among both men and women, elderly family members are highly relied on.

### 4.2 Limitations

The study was not explicitly designed to interrogate participants' perceptions of gender roles of family members in early life nutrition. Therefore, certain knowledge gaps including women's perception of men's role in nutrition need further investigation.

Furthermore, the study has not explored the views of adolescents (under 18), health professionals, community workers or community leaders. Understanding these groups' perceptions might further strengthen the evidence base for developing interventions that both improve nutrition in early life and are gender transformative. Nevertheless, consistency in the study findings between groups suggests the authenticity of the results in this context.

## 5 CONCLUSION

In the context of increasing women's labour force participation, rapid urbanization and industrialization of the South African food systems, we identified changing perceptions of their role as food provides among younger women in Soweto; they are more likely to purchase commercially prepared foods and demonstrate resistance to previous feminized forms of food preparation. In contrast, both men and elderly women support patriarchal gender divisions of labour, which designate mothers as primarily responsible for early life nutrition. To achieve optimal mother and child nutrition, it is imperative that current systems and structures do not reinforce existing power relations but challenge and transform these to become more gender equitable and to be more responsive to the needs of young mothers and their children. Gender transformative policies can ease the disproportionate nutrition burden on women, as well as to support young woman to exercise their agency and claim ownership over nutrition decisions.

## CONFLICTS OF INTEREST

The authors declare that they have no conflicts of interest.

## CONTRIBUTIONS

AE, SAN, MB and KJH designed the research study. AE and SG performed the research and analysed the data. AE, SG, AT and KH wrote the paper. All authors contributed to editing the article and approved the final manuscript.

## Data Availability

Data sharing is not applicable to this article as no new data were created or analysed in this study.
